# Lipopolysaccharide O-antigen profiles of *Helicobacter pylori* strains from Southwest China

**DOI:** 10.1186/s12866-023-03116-0

**Published:** 2023-11-22

**Authors:** Xiaoqiong Tang, Peng Wang, Yalin Shen, Xiaona Song, Mohammed Benghezal, Barry J. Marshall, Hong Tang, Hong Li

**Affiliations:** 1 West China Marshall Research Center for Infectious Diseases, Center of Infectious Diseases, West China Hospital, Sichuan University, Chengdu, 610041 Sichuan China; 2 Division of Infectious Diseases, State Key Laboratory of Biotherapy, West China Hospital, Sichuan University, Chengdu, 610041 Sichuan China; 3 State Key Laboratory of Oral Diseases & National Center for Stomatology & National Clinical Research Center for Oral Diseases &, Department of Orthognathic and TMJ Surgery, West China Hospital of Stomatology, Sichuan University, Chengdu, 610041 Sichuan China; 4 Helicobacter Pylori Research Laboratory, School of Biomedical Sciences, Marshall Centre for Infectious Disease Research and Training, University of Western Australia, Nedlands, Australia

**Keywords:** *Helicobacter pylori*, Lipopolysaccharide, O-antigen, Heptan, Lewis antigen, Antibiotic resistance

## Abstract

**Background:**

*Helicobacter pylori* lipopolysaccharide (LPS) structures vary among strains of different geographic origin. The aim of this study was to characterize the LPS O-antigen profiles of *H. pylori* strains isolated from Southwest China, and to further analyze the association of Lewis antigen expression with clinical outcomes and antibiotic resistance.

**Results:**

A total of 71 *H. pylori* isolates from Southwest China were included for LPS profiling by silver staining and Western blotting after SDS-PAGE electrophoresis. We demonstrated that all the clinical isolates had the conserved lipid A and core-oligosaccharide, whereas the O-antigen domains varied significantly among the isolates. Compared with the common presence of the glucan/heptan moiety in LPS O-antigen structure of European strains, the clinical isolates in this study appeared to lack the glucan/heptan moiety. The expression frequency of Le^x^, Le^y^, Le^a^, and Le^b^ was 66.2% (47/71), 84.5% (60/71), 56.3% (40/71), and 31.0% (22/71), respectively. In total, the expression of type II Le^x^ and/or Le^y^ was observed in 69 (97.2%) isolates, while type I Le^a^ and/or Le^b^ were expressed in 49 (69.0%) isolates. No association of Lewis antigen expression with clinical outcomes or with antibiotic resistance was observed.

**Conclusions:**

*H. pylori* strains from Southwest China tend to produce heptan-deficient LPS and are more likely to express type I Lewis antigens as compared with Western strains. This may suggest that *H. pylori* evolves to change its LPS structure for adaptation to different hosts.

**Supplementary Information:**

The online version contains supplementary material available at 10.1186/s12866-023-03116-0.

## Introduction

*Helicobacter pylori* is a spiral, microaerobic, gram-negative bacterium that chronically colonizes the human gastric mucosa and infects approximately half of the global population [1]. Although most people with *H. pylori* infection have no obvious symptoms, almost all of them have histological chronic active gastritis, which may develop to gastric mucosa atrophy, intestinal metaplasia, and even gastric cancer. *H. pylori* infection has been accepted as the most important etiological factor for gastric cancer [2–5].

The outcomes of chronic *H. pylori* colonization depend on the interaction between bacterial, environmental, and host genetics factors. *H. pylori* lipopolysaccharide (LPS), localized in the outer leaflet of the bacterial outer membrane, plays essential roles in host‒pathogen interactions [6–10]. *H. pylori* LPS consists of three domains, including a hydrophobic lipid A domain embedded in the outer membrane, the outermost O-antigen, and the intermediate core-oligosaccharide domain [7–10]. *H. pylori* lipid A is constitutively modified into a tetra-acylated and mono-phosphorylated structure, which confers *H. pylori* intrinsic resistance to cationic antimicrobial peptides (CAMPs), and the ability to escape Toll-like receptor 4 (TLR4) recognition [7, 11]. Furthermore, *H. pylori* LPS O-antigen usually contains a distal Lewis antigen, which mimics the host Lewis blood group antigens expressed on gastric epithelial cells, camouflaging the bacterium to evade host immune surveillance [7]. Thus, the unique LPS structure plays an important role in chronic colonization of *H. pylori* within the host gastric niche.

**Fig. 1:**
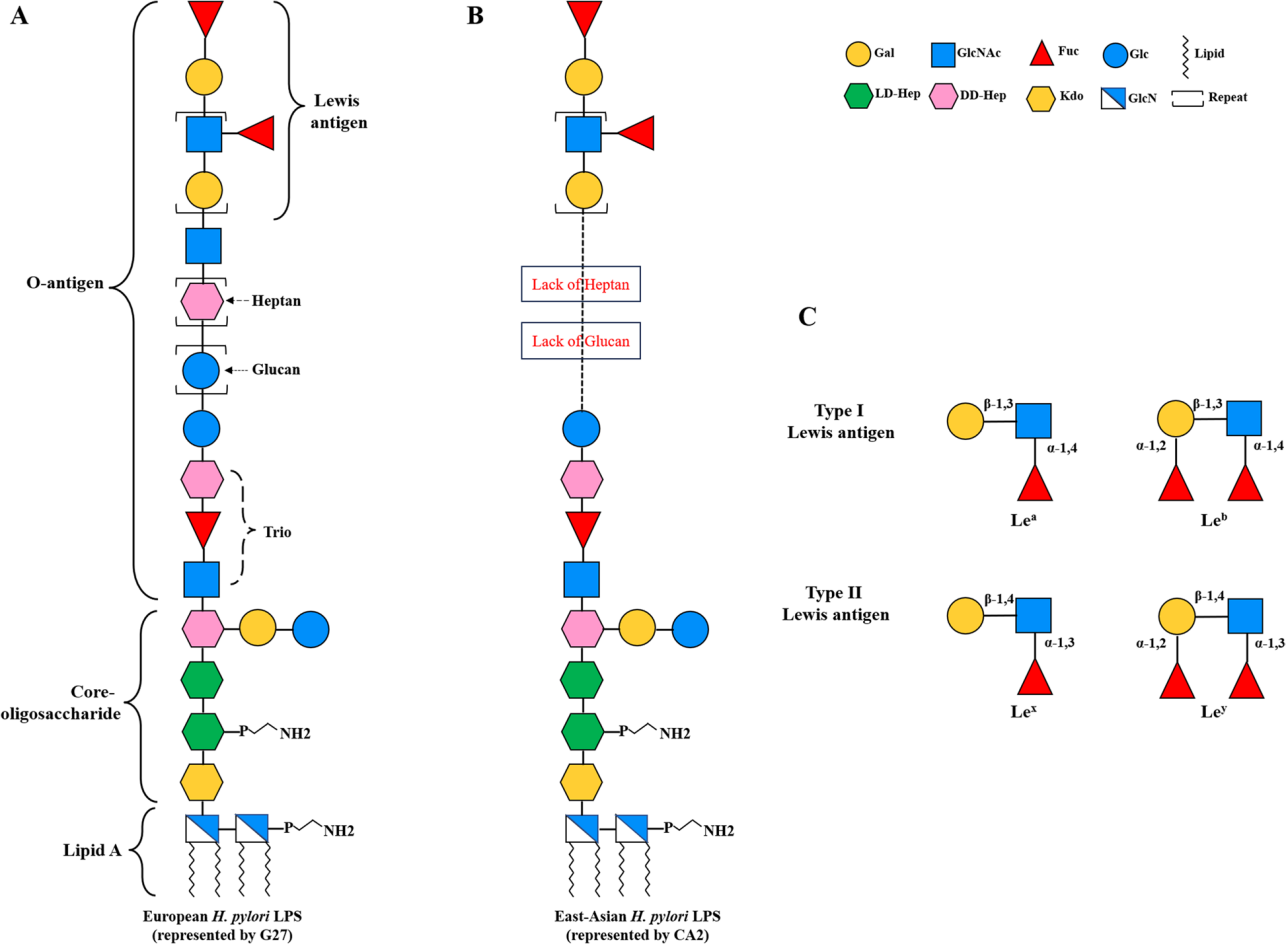
LPS structural differences between Western and East-Asian *H. pylori* strains. **A**: LPS structure of Western *H. pylori* strains (represented by the European reference strain G27) [10]. The lipid A, core-oligosacchardie, and the O-antigen domains are indicated. G27 LPS O-antigen contains the Trio, glucan, heptan, and the distal Lewis antigen; (**B**): LPS structure of East-Asian *H. pylori* strains (represented by the Japanese strain CA2) [10, 12]. CA2 LPS O-antigen contains the Trio and the distal Lewis antigen, but lacks the intermediate glucan and heptan structures; (C): Structures of the type I and type II Lewis antigens in *H. pylori*

Through systematic construction of glycosyltransferase gene mutants combined with LPS structural analysis by mass spectrometry and nuclear magnetic resonance spectroscopy, our group has recently elucidated the LPS structure and related glycosyltransferases in the European *H. pylori* reference strain G27 (Fig. 1A) [8, 10]. Our results redefined the core-oligosaccharide domain as a short conserved hexa-saccharide (KDO-LD-Hep I-LD-Hep II-DD-Hep III-Gal-Glc), whereas the O-antigen is a long linear alignment encompassing not only Lewis antigen but also the conserved trisaccharide (GlcNAc-Fuc-DD-Hep) termed as Trio, the glucan (homopolymer of glucose), and the heptan domains (homopolymer of DD-heptose) [10]. Further comparative genomic analysis among 177 diverse *H. pylori* strains revealed that the glycosyltransferase genes involved in the assembly of Core-Trio-Glc and the distal Lewis antigen motifs are conserved, whereas the heptan glycosyltransferase gene *HP1283* (present in the genome of approximately 80% European strains), is completely absent in the included 74 East-Asian strains, suggesting the absence of heptan in LPS structure of East-Asian strains [8]. This is consistent with a previous study on structural analysis of LPS from 12 East-Asian strains, which has shown the absence of heptan and glucan in LPS from all these strains [12]. Of note, the Japanese strain CA2, one of the 12 East-Asian strains, has been fully sequenced to show the absence of the heptan transferase gene *HP1283* [8] (Fig. 1B). Thus, the most striking difference in O-antigen structure of LPS between Western and East-Asian *H. pylori* strains is the presence and absence of the heptan-glucan structure [8, 9] (compare Fig. 1A and B).

LPS O-antigen in Western and East Asian *H. pylori* strains are also found to be different in the expression of Lewis antigens [12, 13]. *H. pylori* LPS Lewis antigen is composed of a Gal-GlcNAc backbone, which can be divided into type I and type II chains based on the glycosidic linkage (Fig. 1C) [7]. The type I chain is a Gal-(β-1,3)-GlcNAc linkage, which generates Lewis a (Le^a^) with the decoration of Fuc residue to the backbone GlcNAc, and Lewis b (Le^b^) with the further addition of Fuc residue to the backbone Gal, while the type 2 chain is a Gal-(β-1,4)-GlcNAc linkage, which gives rise to Lewis x (Le^x^) and Lewis y (Le^y^) with the decoration of Fuc residues. It has been reported that the majority of Western *H. pylori* strains express type II Lewis antigens, while East-Asian strains are prone to express type I Lewis antigens [12, 14, 15].

LPS structural variations among different strains have been suggested to be related with different clinical outcomes [15, 16], as well as with antibiotic resistance [17–19]. The aim of this study was to characterize the LPS O-antigen profiles of 71 East Asian *H. pylori* strains isolated from Southwest China, and to further analyze the association of Lewis antigen expression with gastric diseases and antibiotic resistance.

## Materials and methods

### *H. pylori* strains and culture conditions

A total of 71 clinical *H. pylori* strains from gastric biopsies previously collected at West China Hospital in Southwest China were included in this study. These clinical strains, together with the reference strain G27, were sub-cultured onto commercial Columbia blood agar (CBA) plates (Autobio, China). The plates were incubated at 37 °C for 24–48 h under microaerobic conditions generated by the Anoxomat Mark-II system (Mart Microbiology B.V., the Netherlands).

### LPS microextraction for silver staining and Western blotting

LPS microextraction was performed based on previously described procedures [20, 21]. Briefly, bacterial cells (OD_600_ = 3) collected from CBA plates were suspended in 100 μL LPS lysis buffer (2% SDS, 4% β-mercaptoethanol, 0.1% bromophenol blue, 10% glycerol, 1 M Tris–HCl (pH 6.8)) and then heated at 100 °C for 10 min. After that, the samples were cooled, and 5 μL proteinase K (PK) (20 mg/ml) was added, incubating the samples in a 55 °C water bath overnight. The obtained LPS samples were run on 15% SDS‒PAGE gels and visualized by silver staining and Western blotting using mouse anti-Le^a^ (1:1500), anti-Le^b^ (1:1500), anti-Le^x^ (1:1500), and anti-Le^y^ (1:1500) (Santa Cruz, USA). After incubation with a secondary rabbit anti-mouse antibody conjugated with peroxidase, HRP conjugates were applied to detect the expression of Lewis antigens.

### Antimicrobial susceptibility testing

We determined the minimum inhibitory concentrations (MICs) for amoxicillin, clarithromycin, levofloxacin, metronidazole, tetracycline and rifampicin using the E-test strips (Liofilchem s.r.l, Italy). In brief, the freshly grown strains were suspended in sterile saline, and the culture suspension with a concentration of 1.0 OD_600_ was evenly inoculated onto commercial Columbia Blood Agar (CBA). Subsequently, the E-test strips were placed firmly onto the inoculated CBA plates and incubated for 3–5 days at 37℃ under microaerobic conditions. According to the recommendations from the European Committee on Antimicrobial Susceptibility Testing (EUCAST), resistance break points for amoxicillin, clarithromycin, levofloxacin, metronidazole, tetracycline and rifampicin were defined as MIC > 0.125 mg/L, > 0.5 mg/L, > 1 mg/L, > 8 mg/L, > 1 mg/L and > 1 mg/L, respectively.

### Statistical analysis

All statistical analyses were performed with SPSS version 18.0 software (SPSS Inc., Chicago, USA). The chi-square test was used to assess the discrepancy in the frequency of endoscopic findings, histological findings and antimicrobial resistance in different Lewis antigen groups. Statistical significance was regarded as* p* < 0.05.

## Results

### Patient demographic and clinical characteristics

A total of 71 clinical *H. pylori* strains consecutively collected from patients of Chinese Han ethnicity were included for characterization of LPS O-antigen profiles and their association with the severity of patient gastric diseases and antibiotic resistance. The patient demographic and clinical characteristics were shown in Table 1 and Table S1. The number of men and women was 36 and 35, respectively, and their mean age was 47.23 years. Chronic gastritis or duodenitis was found in 54 (76.0%) patients, and peptic ulcer in 17 (24.0%) patients. Histological examination of the gastric mucosa showed that non-atrophic gastritis was present in 44 (61.9%) patients, and atrophic gastritis in 27 (38.1%) patients.

**Table 1:** Patient demographic and clinical characteristics

Variables	Number (%) or mean ± SD
**Demographic characteristics**	
Han ethnicity	71 (100.0)
Gender (male/female)	36/35 (50.7/49.3)
Age	47.2 ± 11.5
**Endoscopic findings**	
Chronic gastritis/duodenitis	54 (76.0)
Peptic ulcer	17 (24.0)
**Histological findings**	
Non-atrophic gastritis	44 (61.9)
Atrophic gastritis with/without intestinal metaplasia	27 (38.1)

### Antibiotic resistance profiles of the 71 *H. pylori* clinical isolates

**Fig. 2:**
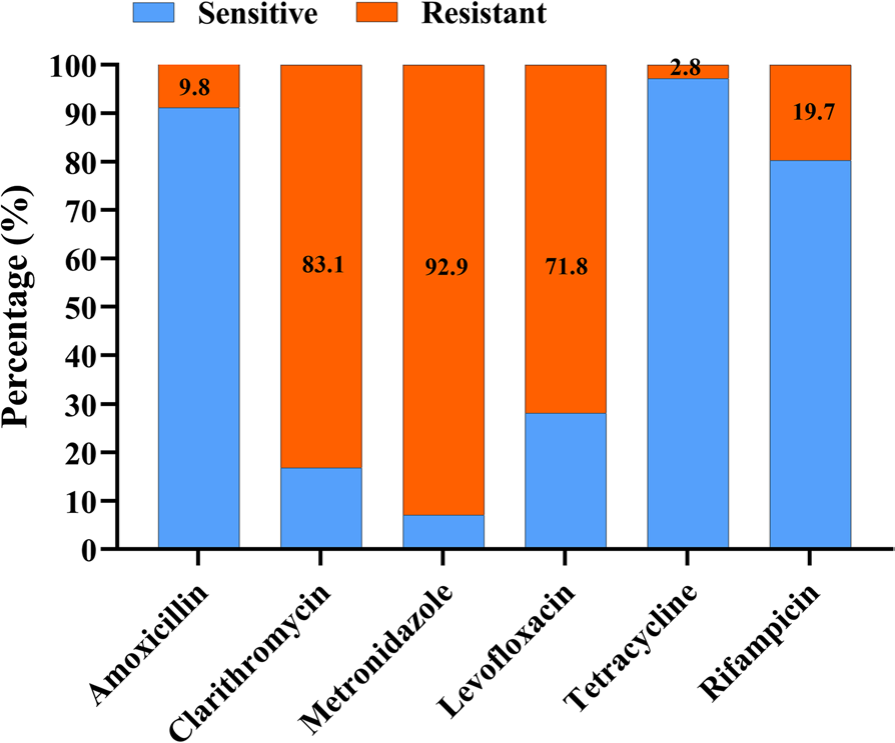
Antibiotic resistance rates of the 71 *H. pylori* clinical isolates

Antibiotic resistance patterns of the 71 clinical strains were profiled by E-test. Antibiotic resistance rates to clarithromycin, metronidazole, and levofloxacin were high, being 83.1%, 92.9%, and 71.8%, respectively. Resistance rates to rifampicin and amoxicillin were 19.7% and 9.8%, respectively, while a negligible 2.8% resistance was found for tetracycline (Fig. 2, Table S1).

### LPS profiles of the 71 *H. pylori* clinical isolates

**Fig. 3:**
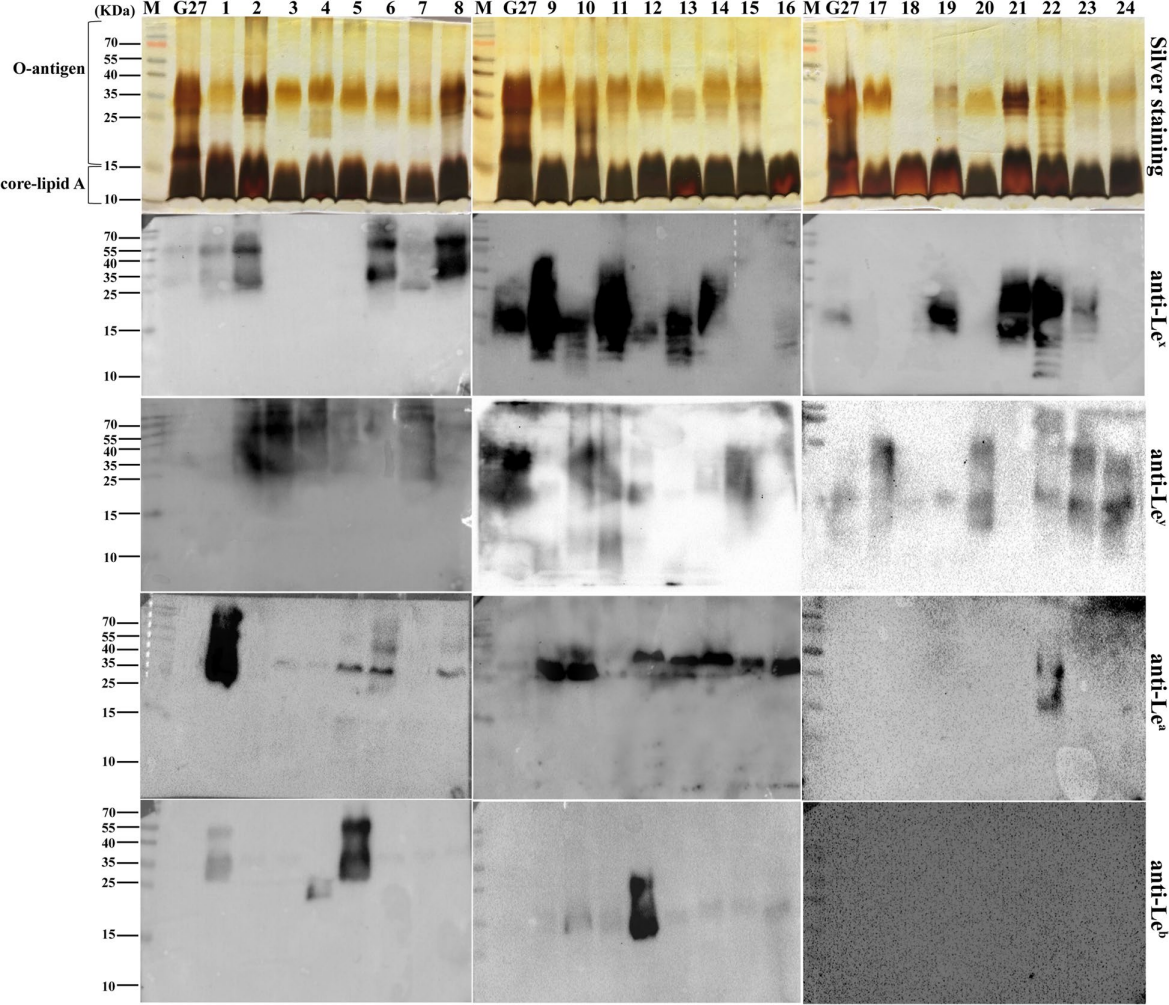
LPS profiles of clinical isolates No. 1–24. LPS samples from G27 wild-type and clinical isolates No. 1–24 were analysed by silver staining and Western blot using anti-Le^x^, anti-Le^y^, anti-Le^a^, and anti-Le^b^

**Fig. 4:**
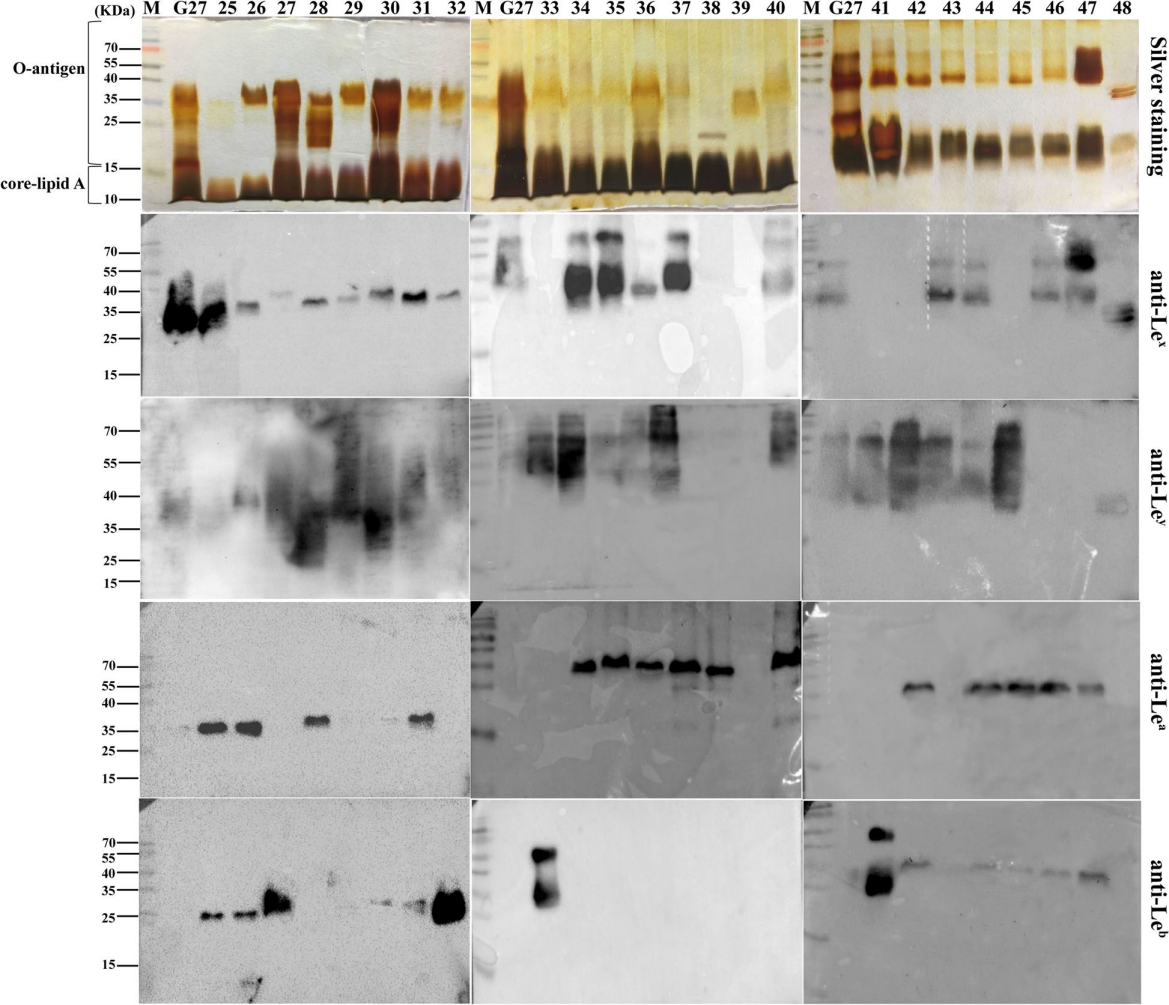
LPS profiles of clinical isolates No. 25–48. LPS samples from G27 wild-type and clinical isolates No. 25–48 were analysed by silver staining and Western blot using anti-Le^x^, anti-Le^y^, anti-Le^a^, and anti-Le^b^

**Fig. 5:**
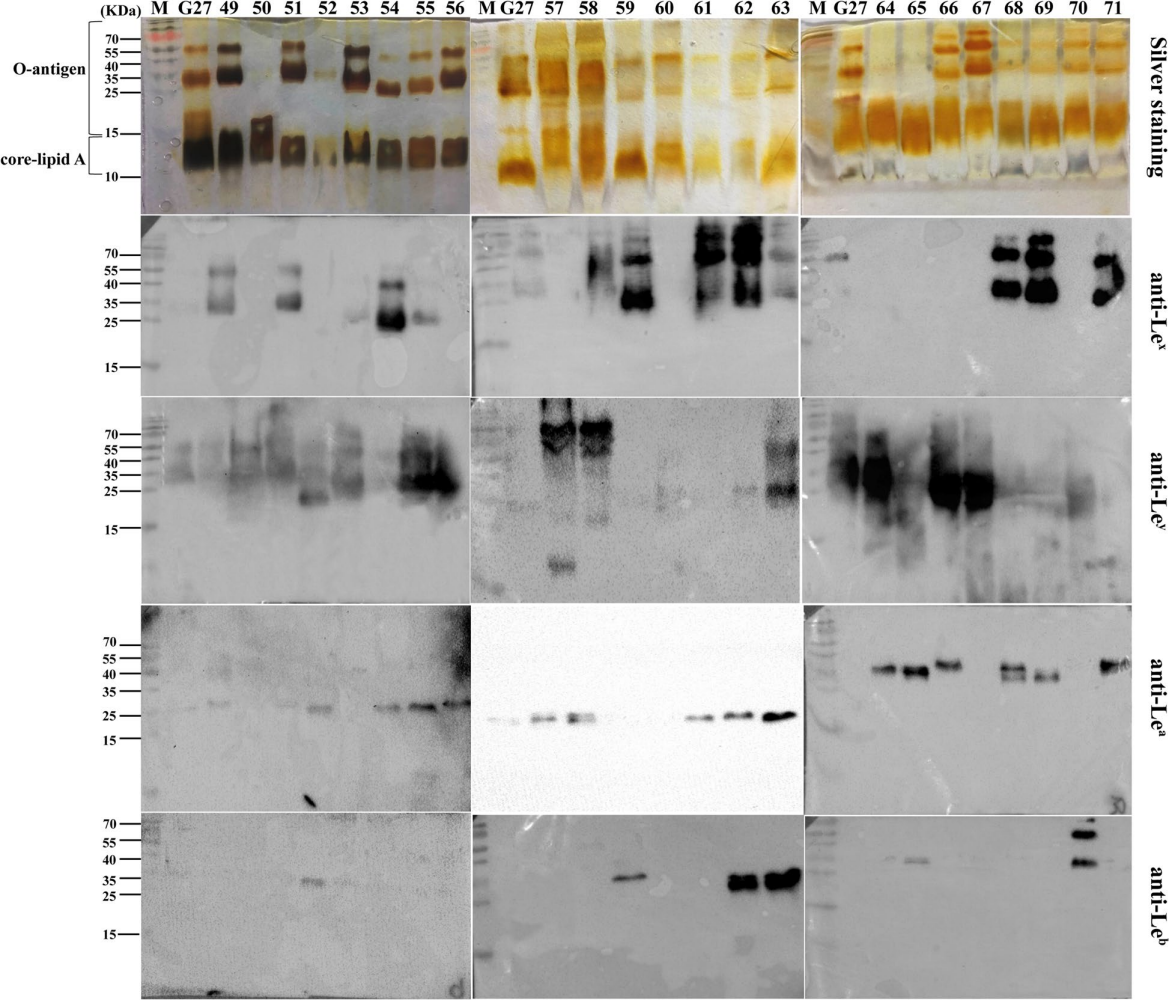
LPS profiles of clinical isolates No. 49–71. LPS samples from G27 wild-type and clinical isolates No. 49–71 were analysed by silver staining and Western blot using anti-Le^x^, anti-Le^y^, anti-Le^a^, and anti-Le^b^

To characterize the LPS profiles, LPS samples extracted from the 71 clinical isolates were resolved on SDS‒PAGE for comparison of LPS general structure by silver staining, and for comparison of Lewis antigen expression by Western bloting (Figs. 3, 4 and 5). As LPS structure of strain G27 has been fully elucidated by our group, LPS extracted from G27 was included as a reference. Of note, G27 LPS is known to harbour type II Le^x^ and Le^y^, but no type I Le^a^ and Le^b^ [8, 10]. For G27 LPS, after SDS-PAGE electrophoresis and silver staining, the stained bands located at 10–20 kDa correspond to LPS lipid A and core-oligosaccharide, while the bands above 20 kDa correspond to O-antigen domain including the Trio, glucan, heptan, and the capping Lewis antigens (Le^x/y^).

Silver staining revealed that G27 and all the 71 clinical isolates had the conserved lipid A and core-oligosaccharide. However, the O-antigen domains varied significantly among the isolates. For example, LPS of isolates 16#, 18#, 25#, and 50# appeared to lack the structure above 35 kDa. LPS of many strains including 41–56# appeared to have a “hollow” in the 15–35 kDa region (Fig. 4, silver staining), which is likely due to the absence of glucan and heptan structures in these strains. In addition, LPS samples from isolates 38# and 48# appeared to be stained uniquely different from the LPS profiles of other isolates (Fig. 4).

Western bloing using anti-Le^x^, and anti-Le^y^, anti-Le^a^, and anti-Le^b^ revealed variations in Lewis antigen expression and chain length among the clinical isolates. Of the 71 *H. pylori* isolates, the expression of Le^x^, Le^y^, Le^a^, and Le^b^ was found to be 66.2% (47/71), 84.5% (60/71), 56.3% (40/71), and 31.0% (22/71), respectively. In total, 97.2% (69/71) of the isolates expressed type II Le^x^ and/or Le^y^, and 69.0% (49/71) expressed type I Le^a^ and/or Le^b^. Interestingly, co-expression of the four Lewis antigens was detected in 6 isolates (12#, 25#, 26#, 31#, 62#, and 63#), while none of the four Lewis antigens was detected in isolate 39# (Figs. 3, 4 and 5, Table S1)).

### Association of Lewis antigen expression with gastric diseases and antibiotic resistance

We further analyzed whether Lewis antigen expression in clinical *H. pylori* strains correlated with clinical outcomes and antibiotic resistance. As shown in Table 2, Lewis antigen expression was neither significantly different between isolates from patients with or without peptic ulcers, nor between isolates from patients with or without atrophic gastritis. Furthermore, our data showed no association between Lewis antigen expression and antibiotic resistance (Table 3).

**Table 2:** The association between Lewis antigen expression and clinical outcomes

Lesion	Le^x^	Le^y^	Le^x^ and/or Le^y^	Le^a^	Le^b^	Le^a^ and/or Le^b^
PU (17)	14 (82.3)	13 (76.5)	17 (100.0)	8 (47.1)	5 (29.4)	10 (58.8)
Non-PU (54)	33 (61.1)	47 (87.0)	52 (96.3)	32 (59.3)	17 (31.5)	40 (74.1)
*p* value	0.19	1.00	1.00	0.55	0.87	0.23
AG (27)	23 (85.2)	21 (77.8)	27 (100.0)	14 (51.8)	8 (29.6)	17 (63.0)
NAG (44)	24 (54.5)	39 (88.6)	42 (95.5)	26 (59.1)	14 (31.8)	32 (72.7)
*p* value	0.01	0.37	0.70	0.89	0.85	0.39

*PU* Peptic ulcer, *NAG* Non-atrophic gastritis, *AG* Atrophic gastritis, *Le*^*x*^ Lewis x antigen, *Le*^*y*^ Lewis y antigen, *Le*^*a*^ Lewis a antigen, *Le*^*b*^ Lewis b antigen

Values are number (percentage)

**Table 3:** The association between Lewis antigen expression and antibiotic resistance

Antibiotics	Le^x^	Le^y^	Le^x^ and/or Le^y^	Le^a^	Le^b^	Le^a^ and/or Le^b^
AML-resistant (7)	2 (28.6)	6 (85.7)	7 (100.0)	4 (57.1)	3 (42.8)	5 (71.4)
AML-susceptible (64)	45 (70.3)	54 (84.3)	62 (96.8)	36 (56.3)	19 (29.7)	46 (71.8)
*p* value	0.07	1.00	1.00	1.00	0.78	1.00
CLR-resistant (59)	39 (66.1)	49 (83.0)	57 (96.6)	33 (55.9)	19 (32.2)	42 (71.2)
CLR-susceptible (12)	8 (66.7)	11 (91.6)	11 (91.6)	7 (58.3)	3 (25.0)	8 (66.7)
*p* value	1.00	0.75	1.00	0.88	0.88	1.00
MTZ-resistant (66)	43 (65.2)	57 (86.3)	64 (97.0)	37 (56.1)	19 (28.8)	45 (68.2)
MTZ-susceptible (5)	4 (80.0)	3 (60.0)	5 (100.0)	3 (60.0)	3 (60.0)	5 (100.0)
*p* value	0.85	0.35	1.00	1.00	0.34	0.32
LEV-resistant (51)	32 (62.7)	44 (86.3)	49 (96.1)	29 (56.9)	16 (31.2)	36 (70.6)
LEV-susceptible (20)	15 (75.0)	16 (80.0)	20 (100.0)	11 (55.0)	6 (30.0)	13 (65.0)
*p* value	0.48	0.77	0.92	0.89	0.91	0.65
TE-resistant (2)	1 (50.0)	2 (100.0)	2 (100.0)	2 (100.0)	1 (50.0)	2 (100.0)
TE-susceptible (69)	46 (66.7)	58 (84.1)	67 (97.1)	38 (55.1)	21 (30.4)	47 (68.1)
*p* value	1.00	1.00	1.00	0.78	1.00	0.85
RIF-resistant (14)	10 (71.4)	10 (71.4)	13 (92.8)	6 (42.8)	2 (14.3)	8 (57.1)
RIF-susceptible (57)	37 (64.9)	50 (87.7)	56 (98.2)	34 (59.6)	20 (35.1)	41 (71.9)
*p* value	0.88	0.27	0.85	0.24	0.47	0.45

*AML* Amoxicillin, *CLR* Clarithromycin, *MTZ* Metronidazole, *LEV* Levofloxacin, *TE* Tetracycline, *RIF* Rifampicin, *Le*^*x*^ Lewis x antigen, *Le*^*y*^ Lewis y antigen, *Le*^*a*^ Lewis a antigen, *Le*^*b*^ Lewis b antigen

## Discussion

*H. pylori* LPS structure is unique and plays important roles in chronic and persistent colonization of *H. pylori* within the host gastric niche [10, 22]. In this study, we evaluated the LPS O-antigen profiles of 71 East Asian *H. pylori* clinical isolates by silver staining and Western bloting. We demonstrated that *H. pylori* LPS lipid A and core-oligosaccharide domains are conserved, while the O-antigen domain varies significantly among the clinical isolates. Furthermore, we demonstrated that both type I and II Lewis antigens are commonly expressed in these isolates, and no association of the Lewis antigen expression frequency with clinical outcomes or antibiotic resistance was found.

Through SDS-PAGE electrophoresis and silver staining of LPS, we observed that the LPS samples extracted from many of the clinical isolates had a “hollow” in the 15–35 kDa region compared with the obvious bands observed in LPS of G27. This “hollow” is likely to be explained by the absence of glucan and heptan motifs in LPS of East-Asian strains [8, 9]. This phenomenon is consistent with the previous LPS structural studies showing the common presence of glucan/heptan structure in Western *H. pylori* strains [12, 18, 23], whereas a complete absence of the heptan moiety in LPS structures of all 12 East-Asian strains [12]. Through comparative genomic analysis, our group has also recently demonstrated the common presence (78%) of the heptan transferase gene *HP1283* in 78 European strains, while a complete absence of the *HP1283* gene in 74 East-Asian strains [8]. The presence and absence of the heptan in LPS structure of Western and East-Asian strains are likely due to *H. pylori* adaptation to different hosts. In addition, the lack of heptan in LPS structure may also be associated with more severe pathogenesis of East-Asian *H. pylori* strains than that of the Westen strains [8].

In the present study, we demonstrated that the type II Le^x^ and/or Le^y^ antigens were dominantly expressed in the Chinese isolates (97.2%), which was comparable to the high expression rate of Le^x/y^ previously reported in both East-Asian and Western clinical isolates (up to 95.4%) [15, 18, 23]. The expression rates of type 1 Le^a^ and/or Le^b^ in our local isolates were 56.3%/31.0%, which were much higher than that in isolates from America and Europe [18, 23, 24]. The expression rates of Le^a^ and Le^b^ in 50 isolates from Greek children [23], 38 isolates from Chileans [24], and 41 isolates from Canadians [18] were 0.02%/22%, 0%/24%, and 0.5%/19.5%, respectively. Thus, our study supports the previous findings that the type II Le^x/y^ antigens are frequently expressed in LPS of both East-Asian and Western strains, whereas a tendency for the expression of type 1 Le^a/b^ antigens in LPS from East-Asian hosts compared with Western populations [12, 15]. The difference of Lewis antigen expression in different populations has been suggested to be related to the host Lewis phenotype, suggesting bacterial host adaptation [25–27].

The molecular mimicry between *H. pylori* LPS Lewis antigens and host Lewis blood-group antigens has been suggested to be involved in the development of autoimmune gastric diseases [7, 22, 28]. It has been previously reported that Lewis antigen expression was significantly higher among isolates from patients who have peptic ulcer disease (PUD) than from those without the PUD [15, 29]. However, there are other studies reporting no association between Lewis antigen expression and gastric lesions [24, 30]. In the present study, we found no association between bacterial Lewis antigen expression and gastric lesions, either. These controversial results may be partly explained by the different Lewis antigen expression measuring methods (ELISA or Western bot) or different antibodies used in these studies. Furthermore, the relatively small number of isolates included is likely to be a small-sample bias for analyzing the association between Lewis antigen expression and gastric lesions. Future studies may need to enroll more subjects and more *H. pylori* isolates from different geographical patients and use the same method to detect Lewis antigen expression to further analyze the relationship between Lewis antigen expression and clinical outcomes.

In recent years, the increasing resistance of *H. pylori* against commonly used antibiotics has posed a great challenge to the success rate of *H. pylori* eradication [4, 31–33]. It is well known that *H. pylori* resistance to clarithromycin and levoflaxacin is mainly due to the point mutations in the 23S rRNA and *gyrA* gene, respectively [34–36]. However, gene mutations can’t well explain *H. pylori* resistance to other antibiotics including metronidazole, amoxicillin. Moreover, there are also clarithromycin and levofloxacin resistant strains without the presence of known point mutations in the 23S rRNA and *gyrA* gene, suggesting the existence of other mechanisms for clarithromycin and levofloxacin resistance [34, 37]. Several previous studies have reported the association of LPS structure with drug resistance [17–19]. Altman et al*.* reported that the expression of type II Lewis antigens was higher in clarithromycin-resistant strains than in clarithromycin-susceptible strains (95.7% vs 77.7%, *p* < 0.05) [18]. However, in the present study, we found no association between the frequency of Lewis antigen expression and resistance to all tested antibiotics (amoxicillin, clarithromycin, levofloxacin, metronidazole, tetracycline, and rifampicin). Our group has recently shown that the deletion of a series of LPS glycosyltransferase genes does not affect *H. pylori* susceptibility to the commonly used anti-H. pylori antibiotics [17].

Our study has limitations. Firstly, the size of the subjects or the number of clinical isolates included in this study was relatively small, which may affect the analysis of association of Lewis antigen expression frequency with antibiotic resistance and clinical outcomes. Secondly, LPS structural analysis by mass spectrometry was not performed for the clinical isolates, and therefore the accurate chemical structures or the absence of the heptan moiety in these Chinese strains can’t be determined.

In summary, our study characterized the LPS profiles of clinical *H. pylori* strains isolated from Southwest China, and analyzed the association of Lewis antigen expression frequency with gastric diseases and antibiotic resistance. We demonstrated that the LPS lipid A and core-oligosaccharide domains are conserved among *H. pylori* strains of different phylogeographic origin, while the LPS O-antigen heptan moiety (commonly present in European strains) appeared to be absent in the clinical isolates. Furthermore, we showed our clinical isolates had a propensity to express more type I Lewis antigens than the Western strains, suggesting bacterial host adaptation. We found no association of Lewis antigen expression with clinical outcomes or with antibiotic resistance.

### Supplementary Information

**Additional file 1: Figure S1. **LPS profiles of clinical isolates No. 1-8. LPS samples from G27 wild-type and clinical isolates were analyzed by silver staining (A); and Western blot using anti-Le^x^ (B), anti-Le^y^ (C), anti-Le^a^ (D), and anti-Le^b^ (E)_._** Figure S2. **LPS profiles of clinical isolates No. 9-16. LPS samples from G27 wild-type and clinical isolates were analyzed by silver staining (A); and Western blot using anti-Le^x^ (B), anti-Le^y^ (C), anti-Le^a^ (D), and anti-Le^b^ (E)_._** Figure S3. **LPS profiles of clinical isolates No. 17-24. LPS samples from G27 wild-type and clinical isolates were analyzed by silver staining (A); and Western blot using anti-Le^x^ (B), anti-Le^y^ (C), anti-Le^a^ (D), and anti-Le^b^ (E)_._** Figure S4. **LPS profiles of clinical isolates No. 25-32. LPS samples from G27 wild-type and clinical isolates were analyzed by silver staining (A); and Western blot using anti-Le^x^ (B), anti-Le^y^ (C), anti-Le^a^ (D), and anti-Le^b^ (E)_._** Figure S5. **LPS profiles of clinical isolates No. 33-40. LPS samples from G27 wild-type and clinical isolates were analyzed by silver staining (A); and Western blot using anti-Le^x^ (B), anti-Le^y^ (C), anti-Le^a^ (D), and anti-Le^b^ (E)_._** Figure S6. **LPS profiles of clinical isolates No. 41-48. LPS samples from G27 wild-type and clinical isolates were analyzed by silver staining (A); and Western blot using anti-Le^x^ (B), anti-Le^y^ (C), anti-Le^a^ (D), and anti-Le^b^ (E)_._** Figure S7. **LPS profiles of clinical isolates No. 49-56. LPS samples from G27 wild-type and clinical isolates were analyzed by silver staining (A); and Western blot using anti-Le^x^ (B), anti-Le^y^ (C), anti-Le^a^ (D), and anti-Le^b^ (E)_._** Figure S8. **LPS profiles of clinical isolates No. 57-63. LPS samples from G27 wild-type and clinical isolates were analyzed by silver staining (A); and Western blot using anti-Le^x^ (B), anti-Le^y^ (C), anti-Le^a^ (D), and anti-Le^b^ (E)_._** Figure S8. **LPS profiles of clinical isolates No. 64-71. LPS samples from G27 wild-type and clinical isolates were analyzed by silver staining (A); and Western blot using anti-Le^x^ (B), anti-Le^y^ (C), anti-Le^a^ (D), and anti-Le^b^ (E)_._

**Additional file 2: Table S1. **Patients characteristics, antimicrobial resistance pattern and Lewis expression among the 71 H. pylori isolates from Southwest China.

## Data Availability

The authors confirm that the data and materials supporting the findings of this study are available within the article and its supplementary materials.
