# Supplementary material for: Lipopolysaccharide O-antigen profiles of Helicobacter pylori strains from Southwest China
Source: BMC Microbiol. 2023 Nov 22;23:360. doi: 10.1186/s12866-023-03116-0 (PMC10664510; doi:10.1186/s12866-023-03116-0)
Supplement: Supplementary file 1 — Additional file 1: Figure S1. LPS profiles of clinical isolates No. 1-8. LPS samples from G27 wild-type and clinical isolates were analyzed by silver staining (A); and Western blot using anti-Lex (B), anti-Ley (C), anti-Lea (D), and anti-Leb (E). Figure S2. LPS profiles of clinical isolates No. 9-16. LPS samples from G27 wild-type and clinical isolates were analyzed by silver staining (A); and Western blot using anti-Lex (B), anti-Ley (C), anti-Lea (D), and anti-Leb (E). Figure S3. LPS profiles of clinical isolates No. 17-24. LPS samples from G27 wild-type and clinical isolates were analyzed by silver staining (A); and Western blot using anti-Lex (B), anti-Ley (C), anti-Lea (D), and anti-Leb (E). Figure S4. LPS profiles of clinical isolates No. 25-32. LPS samples from G27 wild-type and clinical isolates were analyzed by silver staining (A); and Western blot using anti-Lex (B), anti-Ley (C), anti-Lea (D), and anti-Leb (E). Figure S5. LPS profiles of clinical isolates No. 33-40. LPS samples from G27 wild-type and clinical isolates were analyzed by silver staining (A); and Western blot using anti-Lex (B), anti-Ley (C), anti-Lea (D), and anti-Leb (E). Figure S6. LPS profiles of clinical isolates No. 41-48. LPS samples from G27 wild-type and clinical isolates were analyzed by silver staining (A); and Western blot using anti-Lex (B), anti-Ley (C), anti-Lea (D), and anti-Leb (E). Figure S7. LPS profiles of clinical isolates No. 49-56. LPS samples from G27 wild-type and clinical isolates were analyzed by silver staining (A); and Western blot using anti-Lex (B), anti-Ley (C), anti-Lea (D), and anti-Leb (E). Figure S8. LPS profiles of clinical isolates No. 57-63. LPS samples from G27 wild-type and clinical isolates were analyzed by silver staining (A); and Western blot using anti-Lex (B), anti-Ley (C), anti-Lea (D), and anti-Leb (E). Figure S8. LPS profiles of clinical isolates No. 64-71. LPS samples from G27 wild-type and clinical isolates were analyzed by silver staining (A [file 12866_2023_3116_MOESM1_ESM.docx]

**
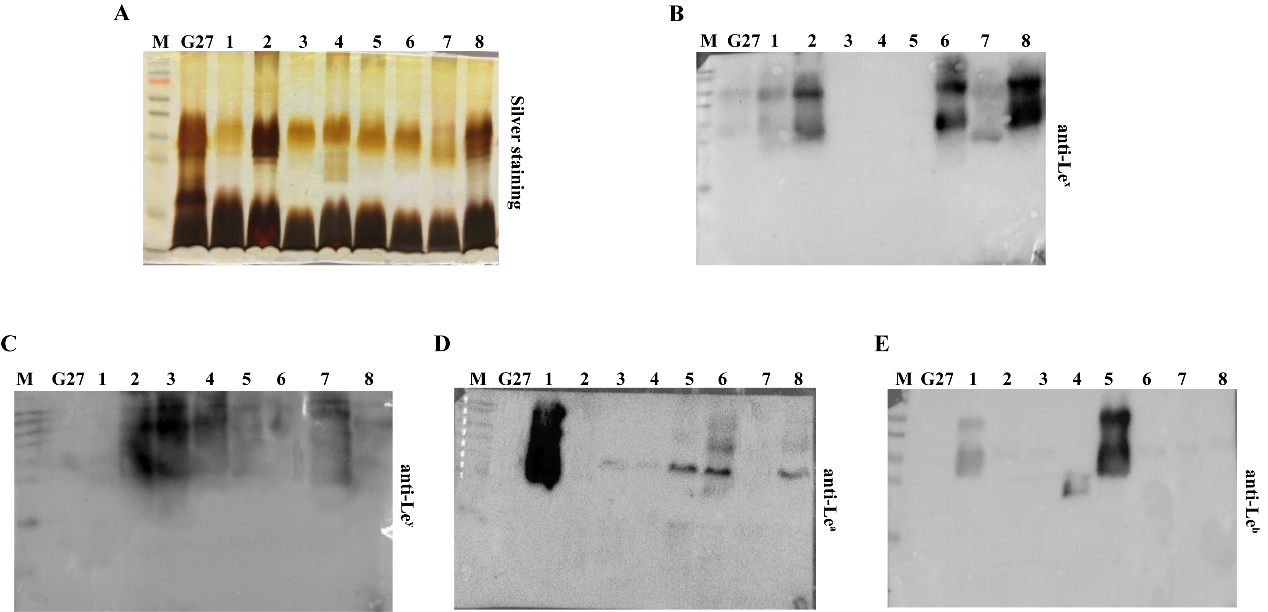
**

**Figure S1. LPS profiles of clinical isolates No. 1-8.** LPS samples from G27 wild-type and clinical isolates were analyzed by silver staining (A); and Western blot using anti-Le^x^ (B), anti-Le^y^ (C), anti-Le^a^ (D), and anti-Le^b^ (E)_._


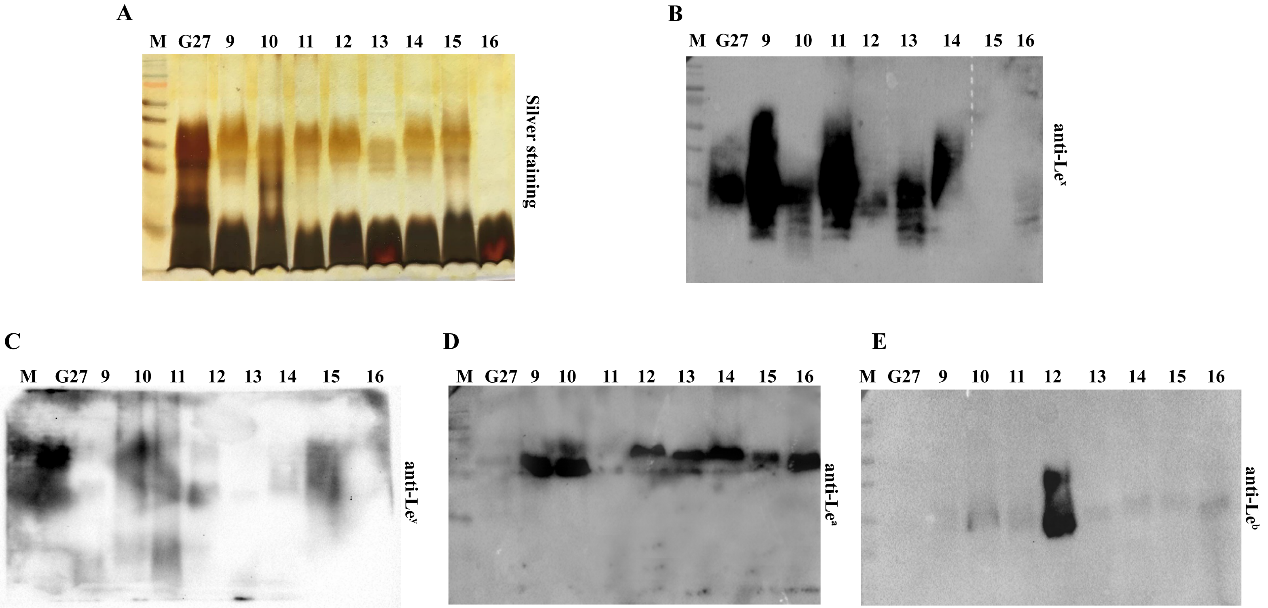


**Figure S2. LPS profiles of clinical isolates No. 9-16.** LPS samples from G27 wild-type and clinical isolates were analyzed by silver staining (A); and Western blot using anti-Le^x^ (B), anti-Le^y^ (C), anti-Le^a^ (D), and anti-Le^b^ (E)_._


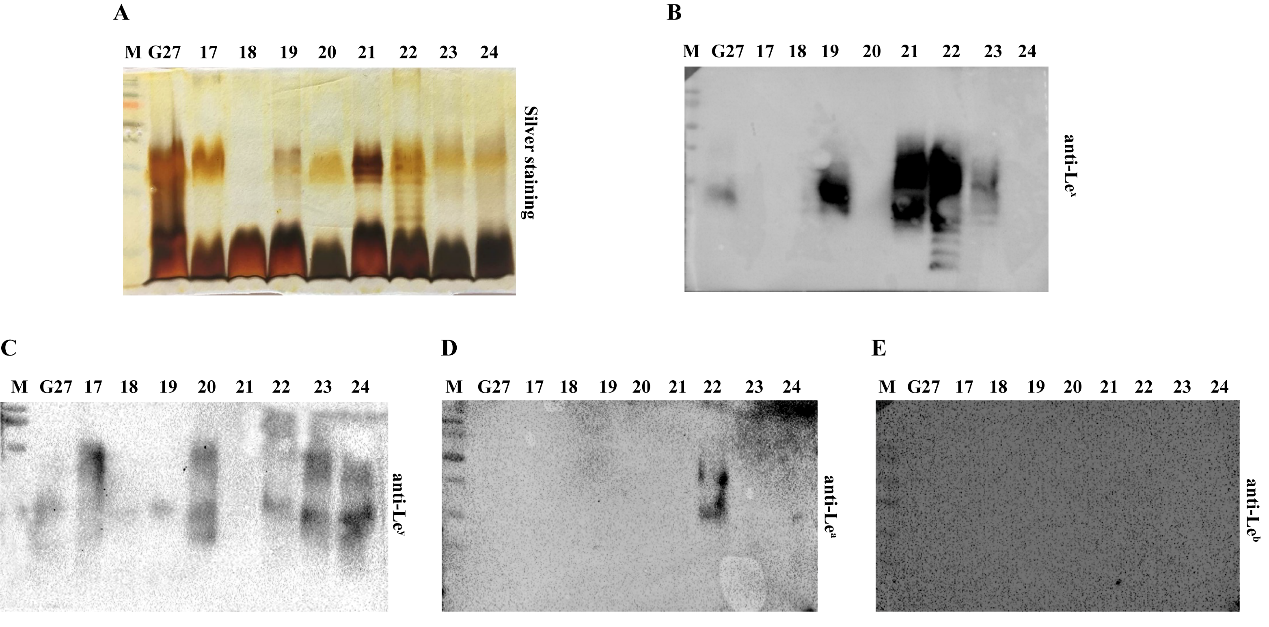


**Figure S3. LPS profiles of clinical isolates No. 17-24.** LPS samples from G27 wild-type and clinical isolates were analyzed by silver staining (A); and Western blot using anti-Le^x^ (B), anti-Le^y^ (C), anti-Le^a^ (D), and anti-Le^b^ (E)_._


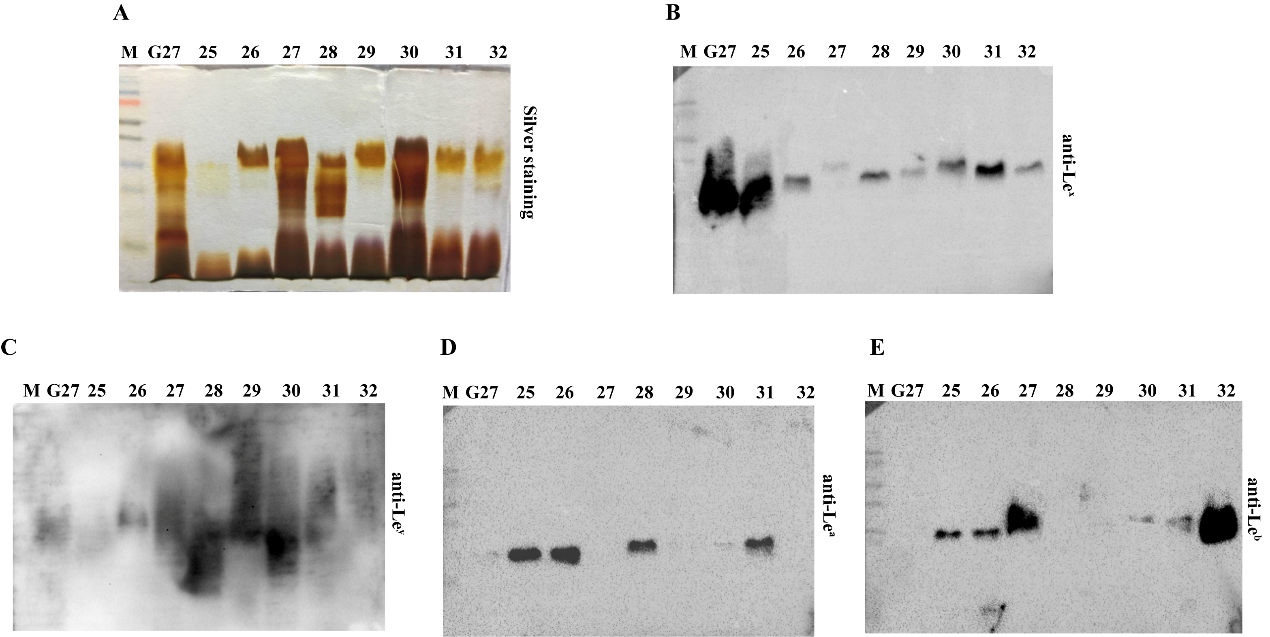


**Figure S4. LPS profiles of clinical isolates No. 25-32.** LPS samples from G27 wild-type and clinical isolates were analyzed by silver staining (A); and Western blot using anti-Le^x^ (B), anti-Le^y^ (C), anti-Le^a^ (D), and anti-Le^b^ (E)_._

**
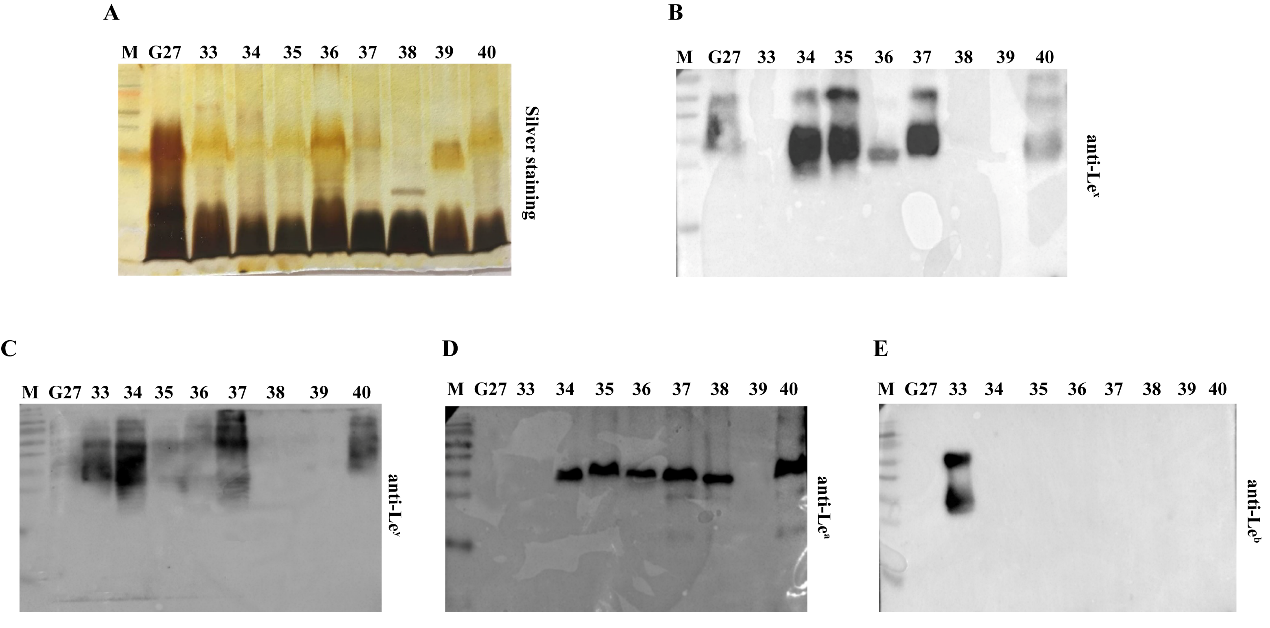
**

**Figure S5. LPS profiles of clinical isolates No. 33-40.** LPS samples from G27 wild-type and clinical isolates were analyzed by silver staining (A); and Western blot using anti-Le^x^ (B), anti-Le^y^ (C), anti-Le^a^ (D), and anti-Le^b^ (E)_._

**
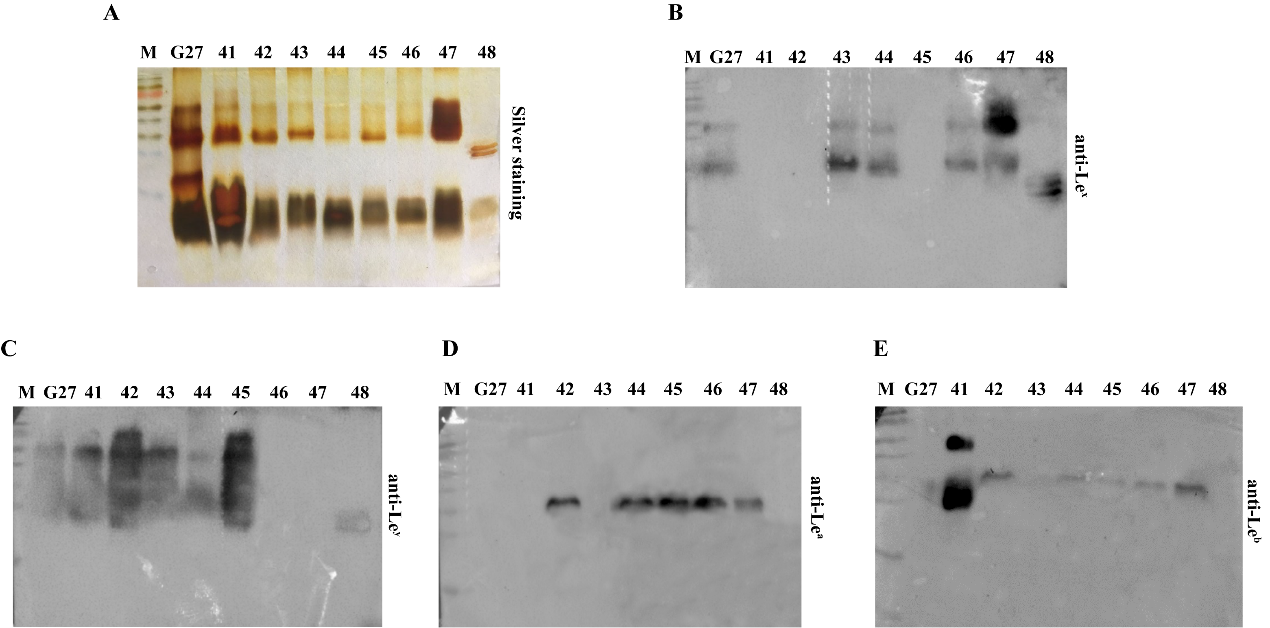
**

**Figure S6. LPS profiles of clinical isolates No. 41-48.** LPS samples from G27 wild-type and clinical isolates were analyzed by silver staining (A); and Western blot using anti-Le^x^ (B), anti-Le^y^ (C), anti-Le^a^ (D), and anti-Le^b^ (E)_._

**
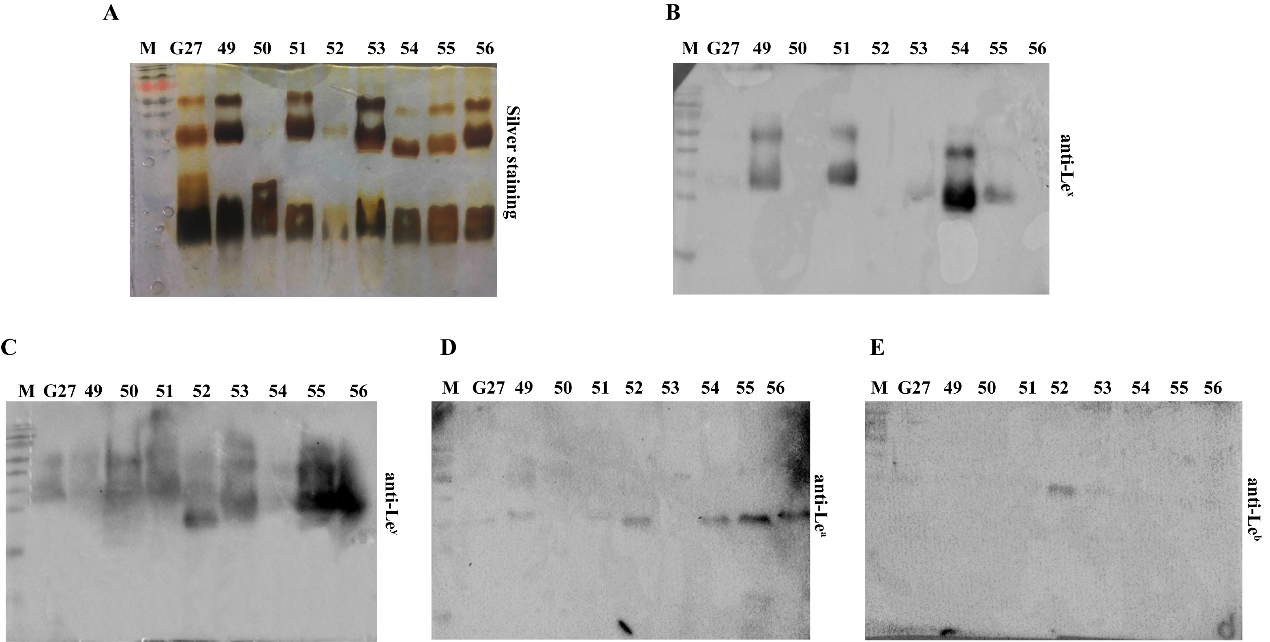
**

**Figure S7. LPS profiles of clinical isolates No. 49-56.** LPS samples from G27 wild-type and clinical isolates were analyzed by silver staining (A); and Western blot using anti-Le^x^ (B), anti-Le^y^ (C), anti-Le^a^ (D), and anti-Le^b^ (E)_._

**
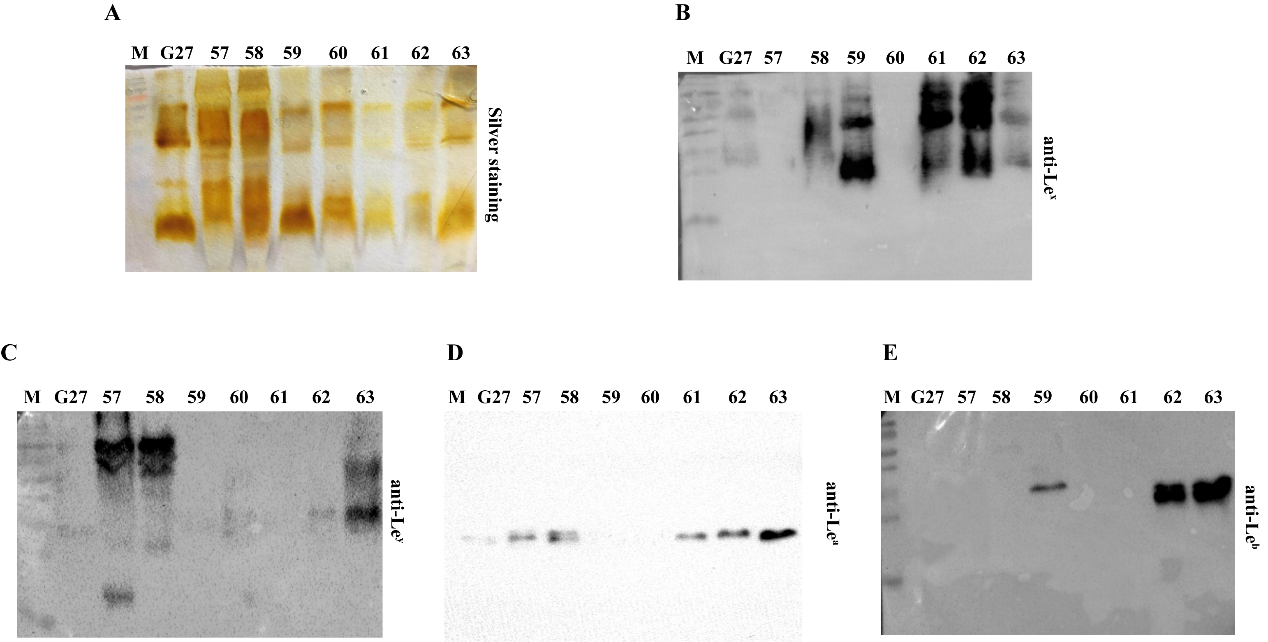
**

**Figure S8. LPS profiles of clinical isolates No. 57-63.** LPS samples from G27 wild-type and clinical isolates were analyzed by silver staining (A); and Western blot using anti-Le^x^ (B), anti-Le^y^ (C), anti-Le^a^ (D), and anti-Le^b^ (E)_._

**
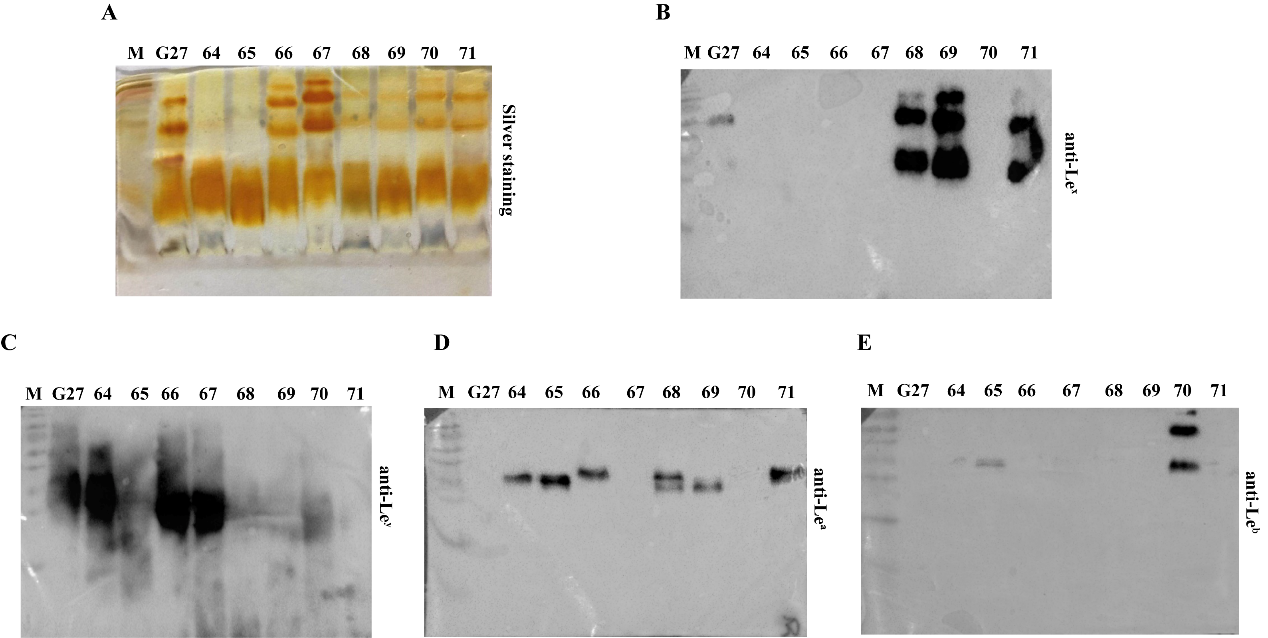
**

**Figure S8. LPS profiles of clinical isolates No. 64-71.** LPS samples from G27 wild-type and clinical isolates were analyzed by silver staining (A); and Western blot using anti-Le^x^ (B), anti-Le^y^ (C), anti-Le^a^ (D), and anti-Le^b^ (E)_._
