# Supplementary material for: Lipopolysaccharide O-antigen profiles of Helicobacter pylori strains from Southwest China
Source: BMC Microbiol. 2023 Nov 22;23:360. doi: 10.1186/s12866-023-03116-0 (PMC10664510; doi:10.1186/s12866-023-03116-0)
Supplement: Supplementary file 2 — Additional file 2: Table S1. Patients characteristics, antimicrobial resistance pattern and Lewis expression among the 71 H. pylori isolates from Southwest China. [file 12866_2023_3116_MOESM2_ESM.docx]

**Table S1.** Patients characteristics, antimicrobial resistance pattern and Lewis expression among the 71 *H. pylori* isolates from Southwest China

| Isolate | Host ethnicity | Host gender | Host age | Endoscopic findings | Histological findings | Antimicrobial susceptibility | | | | | | Lewis antigen | | | | |  |
| --- | --- | --- | --- | --- | --- | --- | --- | --- | --- | --- | --- | --- | --- | --- | --- | --- | --- |
|  |  |  |  |  |  | AML | CLR | MTZ | LEV | TET | RIF | | Le^x^ | Le^y^ | Le^a^ | Le^b^ | |
| 1 | Han | Female | 59 | CG | AG/IM | S | R | R | R | S | S | | + | - | + | + | |
| 2 | Han | Female | 58 | CG+DU | AG/IM | S | S | R | R | S | R | | + | + | - | - | |
| 3 | Han | Male | 49 | CG+CD | NAG | S | R | R | R | S | S | | - | + | - | - | |
| 4 | Han | Male | 43 | CG | NAG | S | R | R | R | S | R | | - | + | - | + | |
| 5 | Han | Male | 46 | CG+GU | NAG | S | R | R | R | S | S | | - | + | + | + | |
| 6 | Han | Male | 52 | CG+DU | NAG | S | R | R | R | S | S | | + | - | + | - | |
| 7 | Han | Male | 53 | CG+DU | AG/IM | S | R | R | R | S | R | | + | + | - | - | |
| 8 | Han | Male | 45 | CG | AG/IM | S | R | R | R | S | S | | + | + | + | - | |
| 9 | Han | Female | 64 | CG | NAG | S | S | S | S | S | S | | + | + | + | - | |
| 10 | Han | Male | 25 | CG+CD | AG/IM | S | S | R | S | S | S | | + | + | + | - | |
| 11 | Han | Male | 40 | CG | AG/IM | S | R | R | R | S | R | | + | + | - | - | |
| 12 | Han | Male | 37 | CG | NAG | S | R | R | S | S | S | | + | + | + | + | |
| 13 | Han | Female | 53 | CG+CD | NAG | S | R | R | R | S | R | | + | + | + | - | |
| 14 | Han | Female | 50 | CG | NAG | S | R | R | S | S | R | | + | + | + | - | |
| 15 | Han | Female | 45 | CG+DU | NAG | S | S | R | R | S | R | | - | + | + | - | |
| 16 | Han | Male | 37 | CG+CD | NAG | S | R | R | R | S | R | | + | - | + | - | |
| 17 | Han | Male | 62 | CG | AG/IM | R | R | R | R | S | S | | - | + | - | - | |
| 18 | Han | Male | 56 | CG+DU | AG/IM | S | S | R | S | S | S | | - | + | - | - | |
| 19 | Han | Male | 41 | CG+DU | AG/IM | S | S | R | S | S | S | | + | + | - | - | |
| 20 | Han | Male | 25 | CG+CD | NAG | S | R | R | S | S | S | | - | + | - | - | |
| 21 | Han | Male | 72 | CG | AG/IM | S | R | R | S | S | R | | + | - | - | - | |
| 22 | Han | Female | 30 | CG | AG/IM | S | S | R | S | S | S | | + | + | + | - | |
| 23 | Han | Female | 51 | CG | NAG | S | S | R | R | S | S | | + | + | - | - | |
| 24 | Han | Male | 52 | CG | NAG | R | R | R | R | S | R | | - | + | - | - | |
| 25 | Han | Female | 36 | CG | AG/IM | S | R | R | R | S | S | | + | + | + | + | |
| 26 | Han | Female | 63 | CG | NAG | S | R | R | R | S | S | | + | + | + | + | |
| 27 | Han | Female | 55 | CG | AG/IM | S | R | R | R | S | R | | + | + | - | + | |
| 28 | Han | Male | 54 | CG+DU | NAG | S | R | R | R | S | S | | + | + | + | - | |
| 29 | Han | Male | 57 | CG+GU | AG/IM | S | R | R | R | S | S | | + | + | - | - | |
| 30 | Han | Male | 64 | CG+GU | AG/IM | S | R | R | R | S | S | | + | + | - | + | |
| 31 | Han | Female | 63 | CG | AG/IM | R | R | R | R | S | S | | + | + | + | + | |
| 32 | Han | Female | 23 | CG | NAG | S | R | R | S | S | S | | + | + | - | + | |
| 33 | Han | Female | 53 | CG | NAG | S | R | R | R | S | S | | - | + | - | + | |
| 34 | Han | Male | 26 | CG | NAG | S | R | R | R | S | S | | + | + | + | - | |
| 35 | Han | Male | 40 | CG | NAG | S | R | R | S | S | S | | + | + | + | - | |
| 36 | Han | Female | 34 | CG | NAG | S | R | R | R | S | S | | + | + | + | - | |
| 37 | Han | Male | 26 | CG | NAG | S | S | S | R | S | S | | + | + | + | - | |
| 38 | Han | Male | 52 | CG+CD | NAG | S | R | R | R | S | R | | - | - | + | - | |
| 39 | Han | Male | 49 | CG+CD | NAG | S | R | R | R | S | S | | - | - | - | - | |
| 40 | Han | Female | 42 | CG | NAG | S | R | R | R | R | S | | + | + | + | - | |
| 41 | Han | Female | 49 | CG+CD | NAG | S | S | S | R | S | S | | - | + | - | + | |
| 42 | Han | Male | 36 | CG+CD | NAG | R | S | R | R | R | S | | - | + | + | + | |
| 43 | Han | Male | 37 | CG | NAG | S | R | R | R | S | S | | + | + | - | - | |
| 44 | Han | Male | 43 | CG+DU | NAG | S | R | R | R | S | S | | + | + | + | - | |
| 45 | Han | Male | 44 | CG+CD | NAG | S | R | R | S | S | S | | - | + | + | + | |
| 46 | Han | Female | 52 | CG | AG/IM | S | R | R | R | S | S | | + | - | + | + | |
| 47 | Han | Female | 56 | CG+DU | AG/IM | S | R | S | S | S | S | | + | - | + | + | |
| 48 | Han | Female | 50 | CG | NAG | S | R | R | S | S | S | | + | + | - | - | |
| 49 | Han | Female | 45 | CG+CD | NAG | S | R | R | R | S | S | | + | + | - | - | |
| 50 | Han | Male | 38 | CG | NAG | S | R | R | S | S | S | | - | + | - | - | |
| 51 | Han | Male | 44 | CG+GU+CD | NAG | S | R | R | R | S | R | | + | + | - | - | |
| 52 | Han | Female | 54 | CG | AG/IM | R | R | R | R | S | S | | - | + | - | + | |
| 53 | Han | Female | 59 | CG+DU | AG/IM | S | R | R | R | S | S | | + | + | - | - | |
| 54 | Han | Female | 48 | CG | NAG | S | R | R | R | S | S | | + | + | - | - | |
| 55 | Han | Female | 68 | CG | AG/IM | S | R | R | S | S | S | | + | + | + | - | |
| 56 | Han | Male | 68 | CG | NAG | S | R | R | R | S | S | | - | + | + | - | |
| 57 | Han | Female | 48 | CG | NAG | R | R | R | R | S | S | | - | + | + | - | |
| 58 | Han | Male | 40 | CG | AG/IM | S | R | R | R | S | S | | + | + | + | - | |
| 59 | Han | Female | 34 | DU | NAG | S | R | S | S | S | S | | + | - | - | + | |
| 60 | Han | Female | 35 | CG+CD | AG/IM | S | R | R | R | S | S | | - | + | - | - | |
| 61 | Han | Male | 66 | CG | AG/IM | R | R | R | R | S | S | | + | - | + | - | |
| 62 | Han | Female | 28 | CG+DU | NAG | S | R | R | R | S | S | | + | + | + | + | |
| 63 | Han | Female | 30 | CG | NAG | S | R | R | S | S | S | | + | + | + | + | |
| 64 | Han | Female | 53 | CG | NAG | S | R | R | R | S | S | | - | + | + | - | |
| 65 | Han | Female | 49 | CG | NAG | S | R | R | R | S | S | | - | + | + | + | |
| 66 | Han | Male | 37 | CG | NAG | S | R | R | R | S | S | | - | + | + | - | |
| 67 | Han | Male | 45 | CG+CD | NAG | S | R | R | S | S | S | | - | + | - | - | |
| 68 | Han | Female | 47 | CG+CD | AG/IM | S | R | R | R | S | S | | + | + | + | - | |
| 69 | Han | Male | 60 | CG | AG/IM | S | R | R | R | S | S | | + | + | + | - | |
| 70 | Han | Female | 39 | CG | NAG | S | R | R | R | S | S | | - | + | - | + | |
| 71 | Han | Female | 45 | GU | AG/IM | S | R | R | S | S | R | | + | - | + | - | |

CG, chronic gastritis; CD, chronic duodenitis; GU, gastric ulcer; DU, duodenal ulcer; NAG, non-atrophic gastritis; AG, atrophic gastritis; IM, intestinal metaplasia; AML, amoxicillin; CLR, clarithromycin; MTZ, metronidazole; LEV, levofloxacin; TET, tetracycline; RIF, rifampicin; S, sensitive; R, resistant.
